# Preparation and Evaluation of a Powdered Rebamipide Mouthwash as In-Hospital Formulation: Considering Dispersion before Use in Patients

**DOI:** 10.3390/pharmaceutics13111848

**Published:** 2021-11-03

**Authors:** Naoko Ishii, Senri Mizobuchi, Yayoi Kawano, Takehisa Hanawa

**Affiliations:** 1 Department of Pharmacy, Kashiwa City Hospital, 1–3 Fuse, Kashiwa 277-0825, Japan; ishii@kashiwacity-hp.or.jp; 2 Faculty of Pharmaceutical Sciences, Tokyo University of Science, 2641 Yamazaki, Noda 278-8510, Japan; 3b18095@ed.tus.ac.jp

**Keywords:** stomatitis, chemotherapy, rebamipide, grinding, dispersibility, dispersion before use, mouthwash, in-hospital formulation

## Abstract

In Japan, rebamipide (RB) mouthwash (RB-MW) for oral mucositis induced by cancer chemotherapy has been prepared using in-hospital formulation. Usually, RB-MW is prepared by dispersing crushed commercial RB tablets in the dispersion medium; however, uniformity is difficult to obtain due to low solubility. The current study aims is to prepare homogenously dispersed formulations using the fine particles of crushed tablets by a method that is convenient for hospital use. Commercial RB tablets were pre-milled at different milling times as “RB-Ts”. A ground mixture was then prepared by co-grinding the RB-Ts with HPC-L or PVP K30 via a benchtop ball milling machine (MM400). The physicochemical properties of samples were evaluated for PXRD, FTIR, turbidity, particle size, and solubility. Although the milling of RB tablets decreased the crystallinity, the length of milling time did not affect them. In contrast, grinding using MM400 significantly decreased RB crystallinity; their PXRD patterns showed a halo, suggesting the amorphization of RB crystals by grinding. Although solubility and turbidity seemed to be affected by the type of polymer rather than the particle size, every ground mixture showed high dispersibility. Thus, grinding the RB-Ts with polymers appeared to be the most promising way to obtain stable dispersion as an in-hospital formulation.

## 1. Introduction

Oral mucositis caused by cancer chemotherapy is reported in 30–40% of patients receiving standard anti-cancer drugs, 70–90% of patients receiving hematopoietic stem cell transplantation, and nearly 100% of patients receiving radiotherapy and anti-cancer drugs [1,2]. Factors that trigger oral mucositis include (1) the generation of active oxygen in the lower oral mucosa due to chemotherapy and radiation therapy and (2) secondary infections associated with malnutrition and decreased immune capacity due to bone marrow suppression [3,4]. In addition, pain due to oral mucositis results in eating and sleep disorders, reduces patients’ quality of life (QOL), and may lead to discontinuation of cancer chemotherapy.

Many methods have been reported for the prevention and treatment of oral mucositis [5,6]. Primary oral care, such as brushing, gargling with oral cleansing agents, and cleaning, is performed [7]. Oral cryotherapy using ice pieces has been employed as symptomatic therapy. For pain, analgesics such as opioids and indomethacin and local anesthetics such as lidocaine are administered [8,9,10,11]. However, these medicines for oral mucositis have not been used as commercial products in Japan. Therefore, original formulations called “in-hospital formulations” have been widely prepared with original methods by pharmacists in hospitals when patients need to have them administrated for the treatment. As these reagents were not available as in-hospital formulations for ethical reasons, commercial products such as tablets have been used for grinding as needed.

As for other commercial products, tablets containing allopurinol, camostat mesylate, and rebamipide (RB), which can remove active oxygen produced by anticancer agents, are commonly used as in-hospital formulations for direct intraoral application in Japan [12,13,14,15,16,17,18]. In particular, RB-containing mouthwash (RB-MW) has been frequently prepared as an in-hospital formulation for oral mucositis due to chemotherapy or radiotherapy in Kashiwa City Hospital. Briefly, the preparation method is that RB commercial tablets containing 300 mg RB are crushed with a tablet crusher, then the compounds are dispersed into 300 mL water with some additives. 

In our previous work, we prescribed RB-MW (3–7 bottles per patient, each 300 mL) to patients with oral mucositis caused by anti-cancer drugs as a clinical trial to assess the course of healing. In addition, we conducted a questionnaire survey in which the patients were asked to respond to queries regarding the status of onset of oral mucositis. If there was an onset, information about the site, size, and degree of pain was collected. It was found that oral mucositis often develops in 7–10 days after the start of chemotherapy and that oral mucositis gradually begins to increase after 2 weeks of radiotherapy [19,20]. However, many patients did not provide their consent to participate in this clinical study due to the requirement to bring home many RB-MW bottles, which resulted in difficulties such as transportation of the mouthwash, the need to store the mouthwash in a cool place, and difficulty in finding a suitable storage location. 

Furthermore, since RB-MW is a suspension consisting of pre-milled RB tablets as dispersoid and polymer solution as a dispersion medium, sedimentation is observed after standing for a few minutes, after which it needs to be shaken before using. However, RB-MW resuspension is difficult due to sediments, including RB, having low solubility; this is considered to cause a lowering in adherence among patients with cancer.

To address these problems, it is necessary to develop a formulation with excellent efficiency and convenient use. We have previously reported that dispersibility of RB-MW can be improved by a wet grinding method using zirconia beads to obtain a reduced particle size of RB with a water-soluble polymer and surfactant [21]. However, since the sample obtained by wet grinding is a suspension, problems such as transportation and storage have not been solved. Therefore, we considered examining a method whereby the formulation can be easily prepared even at medical institutions such as pharmacies, using a powder formulation containing ground RB tablets as the base. It is desirable that this formulation would be powdered during storage and obtain a suspension with high dispersibility when dispersed in water before using.

Thus, it is considered that micronization, including various breakdown and build-up methods, is reasonable for improving dispersibility [22,23,24]. We considered that the breakdown method could be applied in clinical practice because of its simple process. Our previous studies showed that co-grinding RB crystals with various water-soluble polymers and a surfactant results in the transformation of RB crystals into an amorphous form, and that it improves molecular interaction with the polymers. Additionally, the solution showed high dispersion stability [25]. An RB reagent was used as an active pharmaceutical ingredient (API) in the formulations in the studies; however, this was a problem as it was difficult to adapt reagents for use as an in-hospital formulation preparation.

In this study, from the viewpoint of in-hospital formulation preparation, a commercially available RB 100 mg ‘Otsuka’ tablet was pre-milled using a tablet crusher to form a powder. This was followed by adding a water-soluble polymer and co-grinding using a ball mill mixer to prepare a highly dispersible powder. The physicochemical properties of the co-grinding samples were evaluated using powder X-ray diffraction (PXRD) measurements and Fourier transform infrared spectroscopy (FTIR), dispersion stability was assessed by turbidity measurement, and particle size was measured by dynamic light scattering (DLS). Finally, the potential as a preparation method as an “in-hospital formulation” was considered.

## 2. Materials and Methods

### 2.1. Materials

RB 100 mg ‘Otsuka’ tablets were purchased from Otsuka Pharmaceutical Co., Ltd. (lot number: 0K74RT1, expiry date: November 2023, Tokyo, Japan), which consisted of crystalline cellulose, hydroxypropylcellulos, magnesium stearate, hypromellose, polyethylene glycol 6000, and titanium oxide. Hydroxypropyl cellulose (HPC-L), a water-soluble polymer, was provided by Nippon Soda Co., Ltd. (Tokyo, Japan). Polyvinylpyrrolidone (PVPK-30, with a molecular weight of approximately 40,000) was purchased from Nacalai tesque Inc. (Kyoto, Japan). The water-soluble polymer formulations of HPC-L and PVP were used after drying under vacuum at 105 °C. 

### 2.2. Preparation of Co-Grinding Mixtures

This study prepared the ground samples by two steps, listed as “1st step” and “2nd step”.

For the 1st step, a total of 60 RB 100 mg commercial ‘Otsuka’ tablets were milled by the tablet crusher (Labo Milser LM-PLUS Osaka Chemical Co., Osaka, Japan) as “RB-Ts” at different time durations: 30 s (RB-T30), 60 s (RB-T60), and 180 s (RB-T180). 

For the 2nd step, RB-Ts and HPC-L or PVP-K30 at a weight ratio of 1: 5 were physically mixed as “physical mixture, PM” using a vortex mixer (Vortex-Genie 2, Scientific Industries, Inc., New York, NY, USA). Next, co-grinding was performed using a benchtop ball mill mixer MM400 (Retsch, Haan, Germany); i.e., the PM and a stainless steel ball (diameter: 12 mm) were put together in a 10 mL grinding jar; the grinding jar was pre-cooled under liquid nitrogen at −196 °C for 5 min to prevent the sample from overheating. Next, grinding was performed at 30 Hz for 15 min. This process was repeated twice, constituting one cycle. The sample obtained after grinding was a fine particle (ground mixture, GM) (Table 1).

### 2.3. X-ray Powder Diffraction Analysis

The crystallinity of the sample was assessed using a powder X-ray diffractometer (Rigaku Co., Tokyo, Japan). The sample was irradiated with a CuKα_1_ wire through a Ni filter at a voltage of 40 kV and a current of 40 mA. The scanning range was 2*θ* = 5–40°, the scanning speed was 2°/min, and the total count was measured three times.

### 2.4. Fourier Transform Infrared Spectroscopy

Intermolecular interactions of the samples were evaluated using an attenuated total reflectance (ATR) Fourier transform infrared spectrophotometer (FTIR spectrometer, PerkinElmer Co., Ltd., Waltham, MA, USA). The measurement range was 500–4000 cm^−1^.

### 2.5. Preparation of Dispersion for Test Samples 

Each sample was added to ultrapure water to obtain a suspension containing 0.5 mg/mL RB and stirred using a magnetic stirrer for 10 min. The suspension was then processed by ultrasonication for 10 min (at an oscillation frequency of 40 kHz and a high-frequency output of 240 W) and stirred again by a magnetic stirrer for 10 min.

### 2.6. Measurement of Mean Particle Size 

Each sample’s mean particle size and polydispersity index (PDI) were measured using a zeta potential dynamic light scattering analyzer (ELSZ-2000ZS, Otsuka Electronics Co., Ltd., Hirakata, Osaka, Japan). The mean particle size and PDI were analyzed using cumulants fitting analysis after 70 accumulated times. Three batches were prepared for the measurement each sample.

### 2.7. Measurement of RB Solubility

Suspensions of GM containing 0.05 mg/mL as RB concentration were prepared by dispersing the various GMs in ultrapure water and stirring using a magnetic stirrer for 10 min. After that, the suspension was sonicated for 10 min and then stirred using a magnetic stirrer for an additional 10 min. The obtained suspension was centrifuged at 50,000 rpm (240,585× *g*) at 25 °C for 60 min (Himac CP80MX, Hitachi Koki Co., Ltd., Minato-ku, Tokyo, Japan). RB solubility was measured in the supernatant after filtration through a 0.22 μm filter by HPLC. The HPLC system consisted of a pump (PU-22089), UV detector (UV-2075), and column oven (Co-2067) (JASCO Corporation, Hachioji, Tokyo, Japan). HPLC was performed under the following conditions: mobile phase, 0.05 M phosphate buffer solution and acetonitrile (3:1), λmax 326 nm, flow rate 1.0 mL/min, column temperature 30 °C, C18 analytical column (4.6 mm I.D. × 250 mm; Shodex C18M4E, SHOWA DENKO, Column No. K910852).

### 2.8. Evaluation of the Dispersibility of the Suspension

The dispersibility of the sample solution was measured at room temperature (25 °C) using a Turbiscan MA2000 (Formula, Toulouse, France). All samples were suspended in purified water with an RB concentration of 0.2%. Turbiscan was used to evaluate the temporal changes in the concentration gradient of the suspension from the measurement results [26]. The backscattered light was measured by Turbiscan; the near-infrared rays along the flat bottom sample tube were scanned up and down. To observe differences in the sample solution, Transmittance (T) and backscattering (BS) were measured using pulsed near-infrared LEDs at a wavelength of 880 nm to observe differences in the sample solution. T and BS signals were intensity deviations relative to 0. Each sample was mixed by inverting 10 times before being immediately placed on the device. As gargling with the RB-MW is performed 3–6 times a day, it is desirable to maintain dispersibility throughout the day; thus, dispersibility was determined every 10 min for 24 h.

## 3. Results and Discussion

### 3.1. Effect of Grinding on the Crystallinity of Ground Mixtures

The solubility of poorly soluble drugs can be improved by co-grinding or fusing drugs with water-soluble polymers and preparing solid dispersion systems that allow the monomolecular dispersion of the drug between the molecular chains of water-soluble polymers [27,28]. Solid dispersions are usually prepared using reagent grade RB; this study aimed to prepare them as in-hospital formulations, however, regents are not generally used to prepare in-hospital-formulations. Thus, ground RB tablets were used as the principal agents. 

First, RB tablets (total 60 tablets) were milled in a tablet crusher for 30 s (RB-T30), 60 s (RB-T60), and 180 s (RB-T180). During the preliminary experiment, while investigating the grinding time of at least 180 s it was supposed that the motor would likely become damaged due to overheating considering the durability of the machine. Hence, we set the maximum milling time to 180 s. After milling the RB tablets, the sample recovery rates were 93.1, 87.2, and 84.2%, respectively. Therefore, these ground RB tablets were used as samples for the second step, grinding using MM400.

**Figure 1:**
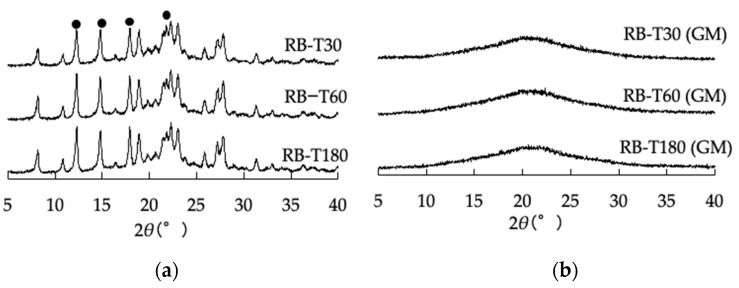
Powder X-ray diffraction patterns of various samples: (**a**) RB-T and (**b**) RB-T (GM).

Figure 1a shows the PXRD patterns of the crushed RB tablets (RB-Ts) at the first step, grinding using a tablet crusher for various durations (30 s, 60 s, and 180 s). It has been reported that RB crystals have characteristic peaks at 2*θ* (◦) = 12.3, 14.7, 17.9, and 21.8 [29]. Diffraction peaks originating from the RB crystals were found at 2*θ* (°) = 12.3, 14.7, 17.9, 22.3 for all samples of RB-T30, RB-T60, and RB-T. Thus, it appears that milling by a tablet crusher did not affect the crystallinity of RB in the samples.

Figure 1b shows the PXRD patterns of ground RB-Ts, i.e., RB-Ts obtained by 1st step grinding which were further ground using a benchtop ball mill (MM400) for 30 min (second step grinding). 

The PXRD patterns of GMs showed a halo pattern, regardless of the duration of the pre-milling time in the tablet crusher. Thus, it was revealed that further grinding of the RB-Ts using MM400 decreased the crystallinity of the RB crystals.

As mentioned above, the diffraction peaks originating from the RB crystals disappeared due to MM400 grinding, which suggests that RB existed in an amorphous state in the GMs. It is well known that the amorphous state has higher free energy and is more energetically unstable than the crystalline form [30]. Therefore, while the drug has improved wettability and dispersibility, it has been considered to easily cause re-crystallization due to its disorderly state [31]. In this study, it was necessary to prevent the re-crystallization of RB because it is stored in the powdered state and intended to form a uniform dispersion when suspended in water immediately before use by patients.

One way to maintain dispersion stability in suspension is by preparing solid dispersions by adding water-soluble polymers, thereby controlling the sedimentation rate of particles during re-dispersion [32,33]. In our previous study, HPCs (HPC-L, HPC-SL, or HPC-SSL) or PVPs (PVPK-30 or PVPK-90) were used as a mixing aid against RB crystals using the dry milling method with MM400. In that study, RB changed to an amorphous form in all of the obtained samples due to grinding; additionally, the preparation of solid dispersion to show intermolecular interaction between RB and HPCs was considered, that is, solid dispersion when composed of RB and HPCs [25]. Therefore, to determine an appropriate water-soluble polymer for the ground RB tablets, HPC-L and PVPK-30 were selected. The weight ratio of RB to HPC-L and PVPK-30 in the compounds was fixed at 1:5 because that ratio showed the smallest particle size of samples in our previous study [25]. 

**Figure 2:**
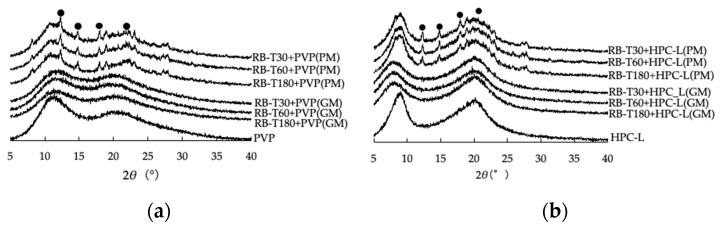
Powder X-ray diffraction patterns of various mixtures: (**a**) with PVPK-30 and (**b**) with HPC-L.

The PXRD pattern of PVPK-30 showed a typical amorphous characteristic, with a broad peak in the vicinity of 2*θ* (°) = 11° (Figure 2a). The peaks of all PMs with PVPK-30 appeared at 2*θ* (°) = 12.33, 14.7, 17.9, 22.3 (Figure 2a). RB crystals have characteristic peaks at 2*θ* (◦) = 12.3, 14.7, 17.9, and 21.8, as described above [29]. The observed peaks of PMs matched the characteristic peaks of RB. Thus, the pre-milling time did not have a significant effect on RB crystallinity. In addition, the PXRD patterns of GMs with PVPK-30 showed halo patterns regardless of the pre-grinding time in the first step and second step (Figure 2a).

The PXRD pattern of HPC-L showed an amorphous characteristic, with a broad peak in the vicinity of 2*θ* (°) = 9° and 20°. The peaks of all PMs with HPC-L appeared at 2*θ* (°) = 12.3, 14.7, 17.9, 18.9, and 27, which were considered to have originated from the RB crystals. Thus, the pre-milling time did not have a significant effect on crystallinity even when HPCs were added. The PXRD patterns of all GMs with HPC-L showed halo patterns, regardless of the pre-milling time (Figure 2b). 

These results show that milling in a tablet crusher did not change RB crystals into an amorphous state, even when the pre-milling time was extended up to 180 s. However, halo patterns were observed when compounds underwent further co-grinding using MM400. It was also observed for all samples obtained using different pre-milling time durations. Furthermore, when grinding with PVPK-30 or HPC-L using MM400, the peaks originated from RB disappeared, and no difference was observed due to the polymer formulation. On the other hand, RB tablets contain many additives, and the effect of these should be considered. The details will be investigated in our future study.

### 3.2. Evaluation of the Intermolecular Interaction by FTIR

**Scheme 1:**
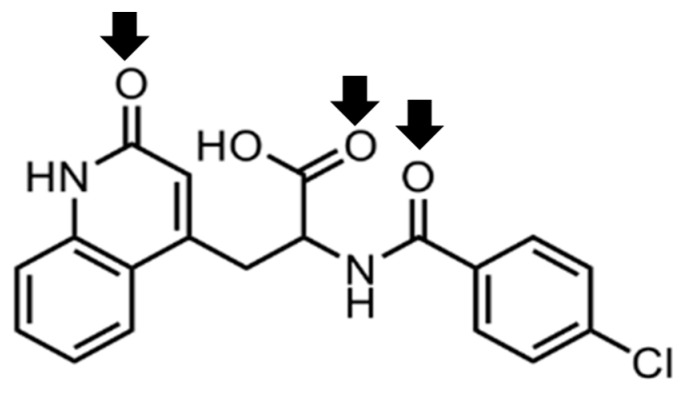
The chemical structure of RB. The black arrows are the carbonyl group focused on in FTIR.

The molecular state of RB in GMs prepared by co-grinding was evaluated by FTIR. RB has three carbonyl groups, which this study focused on. In particular, the absorption spectrum derived from the expansion and contraction oscillation of 1640 cm^−1^ was focused on (Scheme 1) [34].

**Figure 3:**
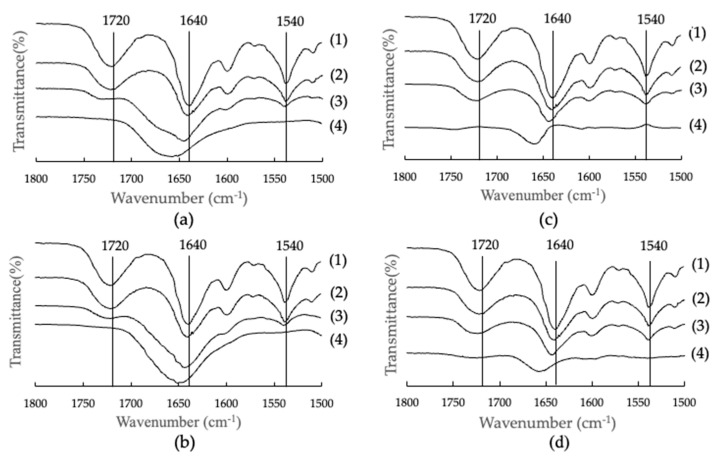
FTIR spectra from 1600 to 1700 cm^−1^ of the various samples. (**a**) RB-T30 + PVPK-30, (**b**) RB-T180 + PVPK-30, (**c**) RB-T30 + HPC-L, and (**d**) RB-T180 + HPC-L. (1) RB crystals, (2) RB-T, (3) PM, (4) GM.

Figure 3 shows the FTIR spectra of RB crystals, samples of RB-T30 and T180 (grinding with a tablet crusher), samples of PMs (RB-T30 or RB-T180 with PVP or HPC-L), and their GMs (grinding with MM400). 

In the RB crystal spectrum, a peak due to carbonyl groups in the amino band (1680–1630 cm^−1^) was observed in the vicinity of 1640 cm^−1^ (Figure 3a–d). 

In the case of RB-T, a peak shift in the vicinity of 1640 cm^−1^ was observed. This was considered to be due to the co-existence of initially added ingredients in the RB tablet, such as HPMC, HPC, and PEG. This peak shift was also clearly observed in the PMs; a peak shift to 1644 cm^−1^ was observed. 

Moreover, a peak shift at 1651 cm^−1^ was observed for RB-T30 + PVP (GM) and at 1658 cm^−1^ for RB-T180 + PVP (GM). Both RB-T30 + HPC-L (GM) and RB-T180 + HPC-L (GM) showed a peak shift at 1658 cm^−1^, suggesting that co-grinding resulted in intermolecular interactions. A peak shift at around 1720 cm^−1^ was also observed. This was considered to be due to the influence of the carbonyl group present in the side chain attached to the carbon at the fourth position of the quinoline nucleus.

Based on the results obtained from the PXRD and FTIR analyses, it was suggested that RB in the GMs interacted with the polymers to form an amorphous solid dispersion (ASD) in which RB exists in a monomolecular state. Intermolecular interaction between RB and water-soluble polymers was observed in the physical mixture of the ground RB tablet and water-soluble polymers. Furthermore, the peak shifts at around 1640 cm^−1^ were also observed clearly in the GMs. 

### 3.3. Pilot Study for Evaluation of the Preservation Stability

**Figure 4:**
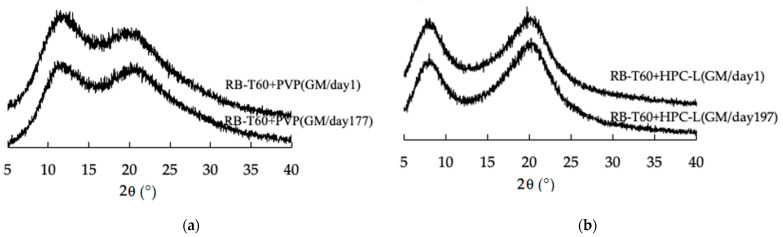
Powder X-ray patterns of samples at storage. (**a**) RB-T60 + PVPK-30 (GM) at day 1 and day 177; (**b**) RB-T60 + HPC-L (GM) at day 1 and day 197.

RB-T60 + PVP (GM) and RB-T60 + HPC-L (GM) were stored with silica gel in a desiccator at room temperature (25 °C) to mimic the storing condition in patients’ homes for approximately six months (Figure 4). On the day of grinding (day 1) and about six months later, the crystallinity of these samples was evaluated using PXRD. The PXRD patterns in either GM had the observed halo patterns. 

There was little change in crystallinity on day 1 or after approximately six months. It was suggested that recrystallization did not occur, even after co-grinding, provided the formulation was stored with silica gels at room temperature. The results suggested that patients could use the RB-MW by dispersing the powder formulation in water by themselves before use at home. However, future studies are needed to investigate the long-term stability of the formulation through acceleration testing and stress testing.

### 3.4. Effect of Grinding on the Particle Size of GMs

**Figure 5:**
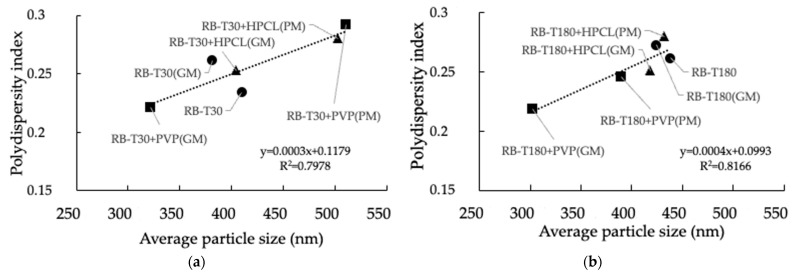
Polydispersity index to average particle size of various samples: (**a**) RB-T30 and (**b**) RB-T180.

The samples were suspended in ultrapure water to determine the particle sizes. Figure 5 shows the relationship between the particle size of the samples pre-milling for 30 s (RB-T30) and 180 s (RB-T180) and their PDIs.

Comparing the pre-milled RB tablets (RB-T30 and BR-T180) with the pre-milled and ball-milled samples [RB-T30(GM) and RB-T180(GM)], the GMs showed a smaller average particle size and lower PDI. Regarding the PMs with HPC-L, the mean particle sizes of GMs became about 100 nm smaller than that of PMs, and the PDI changed to a lower value after 30 s of pre-milling. The PVPK-30 blended sample similarly showed that the average particle size of the GMs was more than 100 nm smaller than that of the PMs for both 30 s and 180 s of pre-milling. Additionally, these results showed that PVPK-30 tended to reduce the particle size more than HPC-L.

The mean particle size and PDI showed a tendency to be proportional in both the RB-T30 and RB-T180 graphs. The coefficient of determination was R^2^ = 0.7978 for RB-T30 and R^2^ = 0.8166 for RB−T180. In other words, these results suggest that the fine particle size obtained by ball-milling is barely affected by pre-grinding time but is affected by the type of polymer selected.

### 3.5. Effect of Grinding on the Solubility of RB

**Figure 6:**
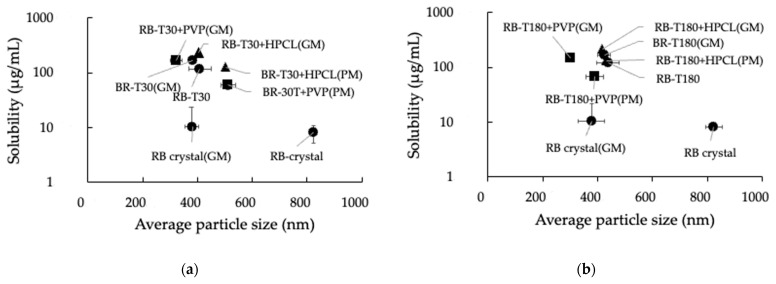
Solubility of rebamipide at various average particle sizes: (**a**) RB−T30 and (**b**) RB−T180.

The solubility of RB in the suspensions of GM containing 0.05 mg/mL as RB concentration was measured. Figure 6 shows the relationship between average particle size and RB solubility. 

As shown in Figure 6, the solubility of the RB crystals was extremely low, and even if the particle size was reduced by MM400 the solubility was hardly improved. In comparison, the solubility of RB-T30 and RB-T180 was higher than the RB crystals. The reason the solubility of RB-T without the subsequent addition of polymer tended to be higher than that of RB crystals can be attributed to the presence of polymers such as hydroxypropyl cellulose (HPC), hypromellose (HPMC), and macrogol 6000 (PEO) as additives in the RB tablets.

It was expected that a smaller average particle size, would be accompanied by a larger the surface area with grinding and a higher solubility; however, the results in this experiment were different. With respect to each pre-milling time in the “First step”, the smallest mean particle size was 332.1 nm and 302.2 nm for RB-T30 + PVPK-30 (GM) and RB-T180 + PVP K-30(GM), respectively. However, the highest solubility was observed for RB-T30 + HPC-L(GM) and RB-T30 + HPC-L(GM) with 229.1 μg/mL and 216.0 μg/mL; in other words, adding HPC-L increased RB solubility. On the other hand, the average particle size of RB-T30 + HPC-L(GM) was 404.7 nm and 418.6 nm, respectively, which was more significant than that of RB-T30 + PVPK-30(GM) and RB-T180 + PVPK-30(GM) despite their solubility being higher than with PVP-K30 added. Among these, the solubility of 216.0 μg/mL of RB−T180 + HPC-L(GM) was the highest, about 27 times higher than the solubility of 8.08 μg/mL RB crystals.

Similar results were reported by Mir et al., who described the solubility of norfloxacin in solid dispersions mixed with PVP and HPC-L and noted that there was a difference in the hydrophilicity of the polymers, which in turn affected the wettability of drugs in solid dispersions [35]. The improved solubility of RB−T180 + HPC-L (GM) observed in this study was also considered to be due to enhanced hydrophilicity due to surface coating by HPC-L. 

These results suggest that the type of polymer influences the increase in the solubility of RB-T in this experimental system, rather than the particle size.

### 3.6. Evaluation of Dispersibility of GMs

In this study, RB-MW was prepared as a formulation to be dispersed for use. Therefore, the dispersion should have good dispersibility when prepared by patients. Accordingly, the dispersibility of the formulations was evaluated using Turbiscan. At the start of the measurement, each sample appeared overall milky white.

**Figure 7:**
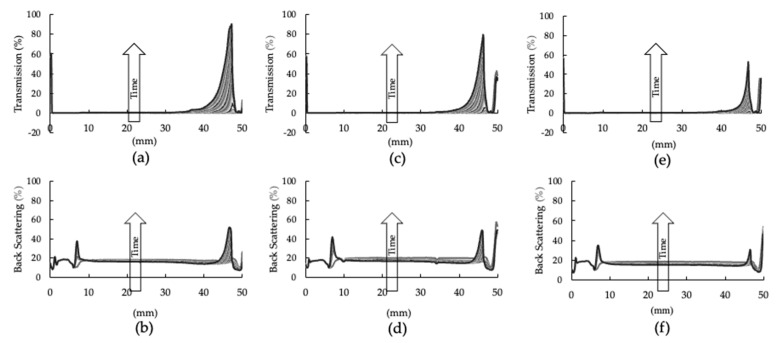
Transmission and backscattering patterns of various suspensions: (**a**) Transmission of RB-T180, (**b**) Back Scattering of RB-T180, (**c**) Transmission of RB−T180 + PVPK-30(PM), (**d**) Back Scattering of RB-T180 + PVPK-30(PM), (**e**) Transmission of RB-T180 + HPC-L(PM), and (**f**) RB-T180 + HPC-L(PM).

**Figure 8:**
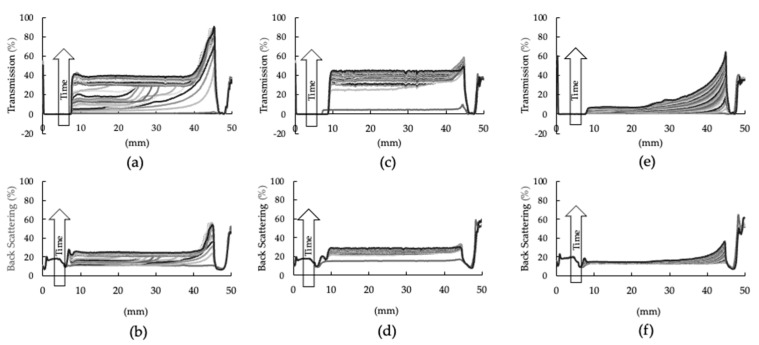
Transmission and backscattering patterns of various suspensions: (**a**) Transmission of RB-T180(GM), (**b**) Back Scattering of RB-T180(GM), (**c**) Transmission of RB-T180 + PVPK-30(GM), (**d**) Back Scattering of RB-T180 + PVPK-30(GM), (**e**) Transmission of RB-T180 + HPC-L(GM), and (**f**) Back Scattering of RB-T180 + HPC-L(GM).

Figure 7 and Figure 8 show the transmission (T) and backscattering (BS) signals of samples suspended in purified water. 

Figure 7 shows the results for RB-T180, RB-T180 + PVP (PM), and RB-T180 + HPG-L (PM). From Figure 7a,b, T and BS increased at upper layer of the sample tube, and it can be seen that the permeability of the supernatant increases over time and becomes partially transparent in samples without the physical mixing of polymers. In samples with physically mixed polymers, the increase in T and BS was smaller than in RB-T180 (Figure 7c–f). In particular, the percentage increase in RB-T180 + HPC-L(PM) was low (Figure 7e,f). In addition, BS was increased at the bottom of all test tubes. The samples in this study were commercially available film-coated tablets. Sieving in pre-milling (First step) removed the film covering the tablets, but some additives remained. The RB tablets included crystalline cellulose as an additive, which is insoluble in water. Therefore, the reason for the sedimentation was considered to be the effect of insoluble additives such as crystalline cellulose.

Figure 8 shows the results for RB-T180, RB-T180 + PVP (GM), and RB-T180 + HPC-L (GM). The T and BS of RB-T180(GM) increased with time (Figure 8a,b). For RB-T180 + PVPK-30 (GM), T and BS both showed an overall increase after 10 min; however, no significant change was observed until 24 h later (Figure 8c,d). In addition, no significant peaks were observed in the upper layer of the test tube. For RB-T180 + HPC-L(GM), peak T was observed in the upper layer, but the backscattered light was largely suppressed (Figure 8e,f). As in the other samples, aggregation was observed, though to a lesser degree.

The above results show that HPC-L is the most helpful polymer for improving the dispersion of RB-T.

## 4. Conclusions

In this study, we presented a method for RB-MW preparation using commercially available RB tablets by grinding using a tablet crusher as a pre-milling process before co-grinding with HPC-L or PVP using a ball mill. 

In addition to, the improvement of the formulation’s dispersibility, it was considered desirable that patients be able to disperse it before use. Although RB tablets contain additives, co-grinding them with a water-soluble polymer formulation can amorphize the RB crystals. Furthermore, FTIR measurements revealed the interactions between molecules, suggesting that they were stable ASDs. In addition, it was found that the solubility of RB was not affected by particle size but was affected by the polymer selected for the physical mixtures and by grinding with a ball mill.

On the other hand, the dispersibility of PMs was better than GMs; the agglomeration of GM due to its small particle size was considered to prevent the improvement in dispersibility. However, HPC-L tended to be less affected by agglomeration. These results suggest that it is essential to select an effective polymer in order to improve the dispersibility of insoluble drugs. Therefore, the commercial RB tablets studied here can be used as a raw material for in-hospital formulation by physically mixing and grinding them with water-soluble polymers. Furthermore, it was considered that the method we introduced to prepare RB-MW within a short time might be reasonable for preparation of in-hospital formulations.

Nanoparticles, with a mean particle size of 200 nm or less, have been reported to diffuse the mucin layer in the oral cavity [36]. Although there no samples reached less than 200 nm in this experimental method, investigation of the use of polymers with a different molecular weight may need to be performed in a future study. Additionally, the evaluation of the stability of the formulations through acceleration testing and stress testing for long-term storage is important for application as an in-hospital formulation; thus, this should also be evaluated in a future study.

## Figures and Tables

**Table 1:** Formulations of various samples.

Sample	Mixing Weight Ratio			Pre-Milling Time (Second)	Ground Time (Min)
	RB-T *^1^	PVPK-30	HPC-L		
RB-T30	1	−	−	30	−
RB-T30 (GM) *^2^	1	−	−		30
RB-T30 + PVPK-30 (PM) *^3^	1	5	−		−
RB-T30 + PVP K-30 (GM)	1	5	−		30
RB-T30 + HPC-L (PM)	1	−	5		−
RB-T30 + HPC-L (GM)	1	−	5		30
RB-T60	1	−	−	60	−
RB-T60 (GM)	1	−	−		30
RB-T60 + PVPK-30 (PM)	1	5	−		−
RB-T60 + PVPK-30 (GM)	1	5	−		30
RB-T60 + HPC-L (PM)	1	−	5		−
RB-T60 + HPC-L (GM)	1	−	5		30
RB-T180	1	−	−	180	−
RB-T180 (GM)	1	−	−		30
RB-T180 + PVPK-30 (PM)	1	5	−		−
RB-T180 + PVPK-30 (GM)	1	5	−		30
RB-T180 + HPC-L (PM)	1	−	5		−
RB-T180 + HPC-L (GM)	1	−	5		30

*^1^ RB-T is a powder of pre-milled rebamipide tablets. *^2^ GM is an abbreviation for ground mixture. *^3^ PM is an abbreviation for physical mixture.
